# Supplementary material for: Highly efficient intercellular spreading of protein misfolding mediated by viral ligand-receptor interactions
Source: Nat Commun. 2021 Oct 19;12:5739. doi: 10.1038/s41467-021-25855-2 (PMC8526834; doi:10.1038/s41467-021-25855-2)
Supplement: Supplementary file 1 — Supplementary Information [file 41467_2021_25855_MOESM1_ESM.docx]

**SUPPLEMENTARY INFORMATION**

Highly efficient intercellular spreading of protein misfolding

mediated by viral ligand - receptor interactions

Shu Liu^1, 2^, André Hossinger^1^, Stefanie-Elisabeth Heumüller^1^, Annika Hornberger^1^, Oleksandra Buravlova^1^, Katerina Konstantoulea^3, 4^, Stephan A. Müller^5, 6^, Lydia Paulsen^1^, Frederic Rousseau^3, 4^, Joost Schymkowitz^3, 4^, Stefan F. Lichtenthaler^5, 6, 7^, Manuela Neumann^8, 9^, Philip Denner^1^ and Ina M. Vorberg^1, 10*^

^1^German Center for Neurodegenerative Diseases Bonn (DZNE), Venusberg Campus 1/ 99, 53127 Bonn, Germany

^2^present address: German Federal Institute for Risk Assessment (BfR), German Centre for the Protection of Laboratory Animals (Bf3R), Max-Dohrn-Straße 8-10, 10589 Berlin, Germany

^3^VIB Center for Brain and Disease Research, Leuven, Belgium

^4^Switch Laboratory, Department of Cellular and Molecular Medicine, KU Leuven, Leuven, Belgium

^5^German Center for Neurodegenerative Diseases (DZNE), Munich, Germany

^6^Neuroproteomics, School of Medicine, Klinikum rechts der Isar, Technical University of Munich, 81675 Munich, Germany

^7^Munich Cluster for Systems Neurology (SyNergy), Munich, Germany

^8^Department of Neuropathology, University Hospital Tübingen, Tübingen, Germany

^9^Molecular Neuropathology of Neurodegenerative Diseases, German Center for Neurodegenerative Diseases (DZNE), Tübingen, Germany

^10^Rheinische Friedrich-Wilhelms-Universität Bonn, Venusberg Campus 1, 53127 Bonn, Germany

**Supplementary Tables**

Supplementary Table 1.: Human Brain Samples

| Patient No. | Age at death | Sex | NP-Diagnosis | ABC score* | Brain region |
| --- | --- | --- | --- | --- | --- |
| 1 | 65 | M | AD | A3, B3, C3 | frontal cortex |
| 2 | 63 | F | CBD | A2, Bx, C1 | frontal cortex |
| 3 | 77 | M | PSP | A1, B1, C1 | pons |
| 4 | 56 | M | FTLD-tau (MAPT IVS10+3 G>A) | A0, Bx, C0 | frontal cortex |
| 5 | 79 | F | Control | A1, B1, C0 | frontal cortex |

*ABC score according to the National Institute of Aging-Alzheimer’s association guidelines ^1^.

Supplementary Table 2.: Primer for VSV-G mutagenesis

| Name | Sequence |
| --- | --- |
| VSV-G W72A (Forward) | 5’-AGCAGACGGTgcgATGTGTCATG-3’ |
| VSV-G W72A (Reverse) | 5’-TGAATAGCCTTGTGACTC-3’ |
| VSV-G K47A (Forward) | 5’-CAAAATGCCCgcgAGTCACAAGGC-3’ |
| VSV-G K47A (Reverse) | 5’-ACTTGTATGGCTGTGCCT-3’ |

**Supplementary Figures**


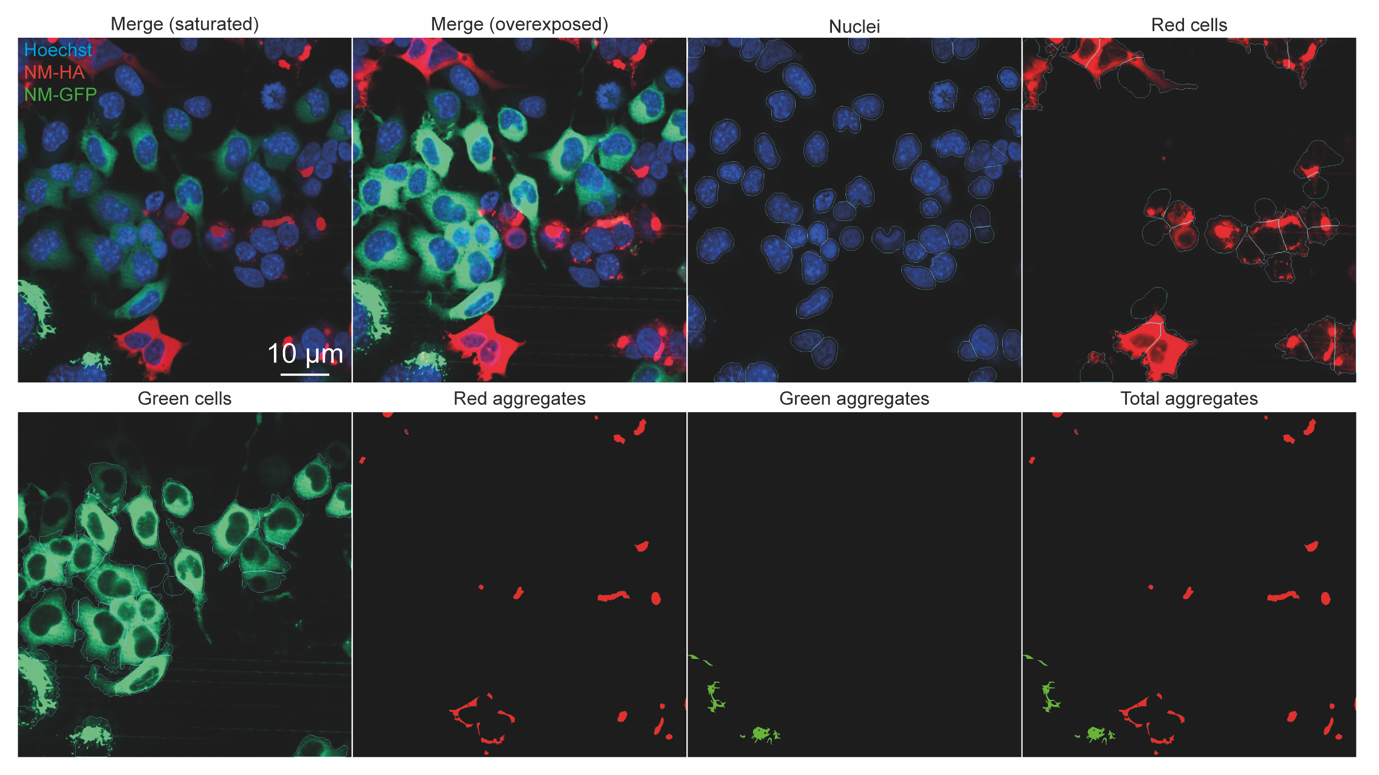


**Suppl. figure 1.** **Automated image analysis exemplified for the coculture assay.** Donor cells stably propagating aggregated NM-HA (red) and recipient cells expressing soluble NM-GFP were seeded on 384 well plates. 12-24 h post coculture, cells were fixed and nuclei were stained with Hoechst. For some experiments, NM-HA was stained with anti-HA antibody. Maximal projection images (Z-stacks) were automatically captured using the CellVoyager6000 confocal microscope with a 20 x water immersion objective. Per well, a minimum of 16 random fields were imaged. Automated imaged analysis detected nuclei, red and green cells and green cells with green aggregates.


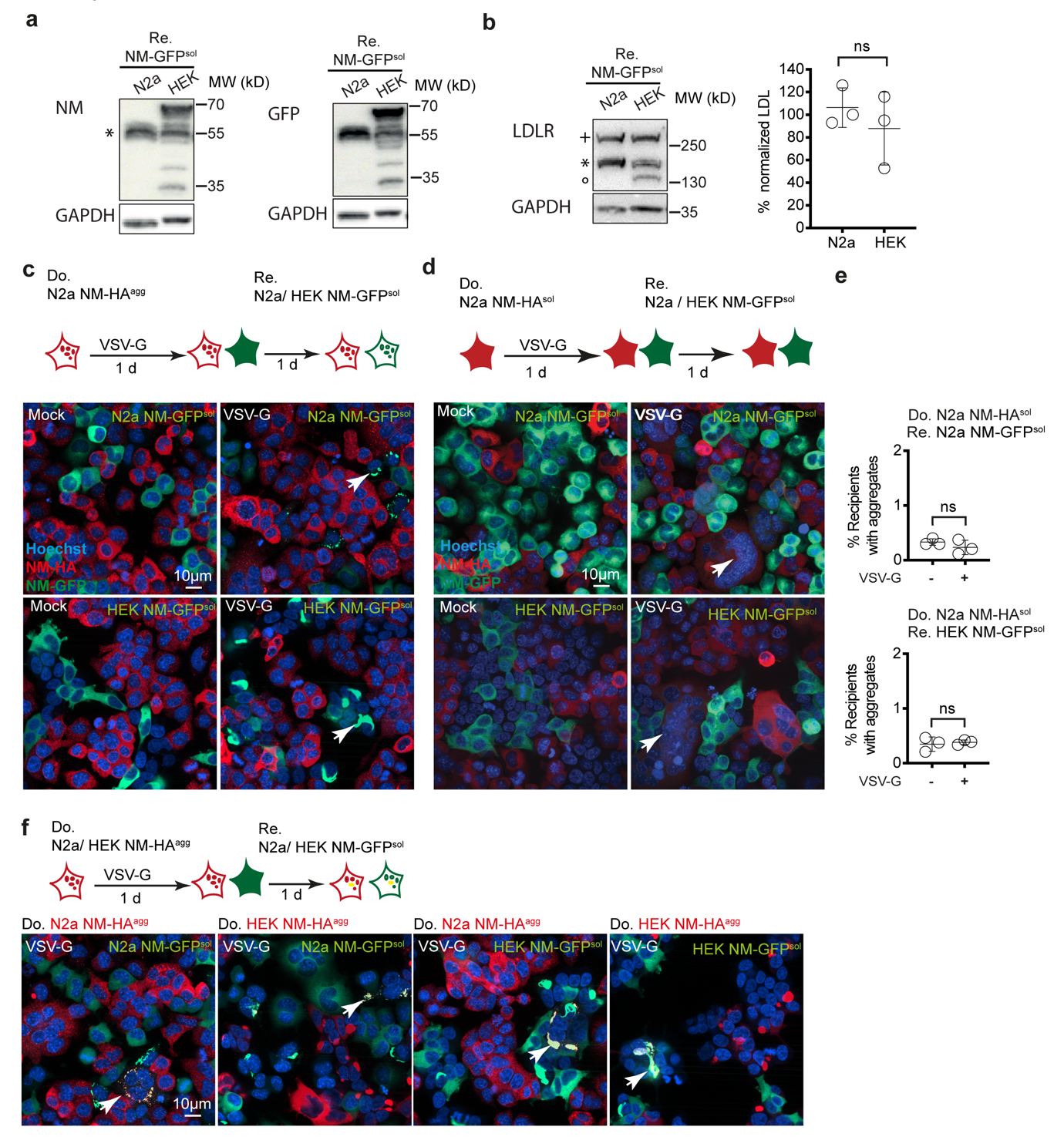


**Suppl. figure 2. Coculture of HEK and N2a cells expressing NM-HA.** a. Western blot analysis of NM expression in HEK and N2a cells expressing soluble NM-GFP. NM-GFP was detected using an antibody against the M domain of NM ^2^ and anti-GFP antibodies. The expected size is indicated by a star. Note that in HEK cells, NM-GFP is partially proteolytically cleaved, resulting in lower molecular weight bands and a 70 kD band, likely representing dimeric proteolytic cleavage products detected by both anti-NM and anti-GFP antibodies. b. Western blot analysis and quantification of LDL receptor expression in recipient HEK and N2a NM-GFP^sol^ cells. The expected size of glycosylated LDL receptor ^3^ is indicated by a star, unglycosylated LDL by an open circle. Dimers ^3^ are indicated by a cross. To quantify, all antibody-positive bands were included. Mean expression levels in N2a cells were set to 100 %. c. Donor N2a NM-HA^agg^ cells, Mock or VSV-G transfected, cocultured with either N2a or HEK cells expressing soluble NM-GFP. NM-HA was stained using anti-HA antibodies. Arrowheads indicate induced NM-GFP aggregates. d. Donor N2a NM-HA^sol^ cells, Mock or VSV-G transfected, cocultured with either N2a or HEK cells expressing soluble NM-GFP. Arrowheads indicate multinucleated cells. e. Quantitative analysis of automated confocal images of (d). Shown are the percentages of cells detected as aggregate-positive by the algorithm. f. Experimental design. Donor N2a and HEK NM-HA^agg^ cells were transfected with VSV-G plasmid or Mock transfected. The following day, donor cells were cocultured with recipient N2a or HEK cells expressing NM-GFP^sol^. Syncytia formation and/or NM-GFP/ NM-HA costained aggregates indicate fusogenic activity of VSV-G. Arrowheads indicate multinucleated cells and/or cells with protein aggregates positive for both HA and GFP. All data are shown as the means ± SD from three (b, e) replicate cell cultures. Three (b, e) independent experiments were carried out with similar results. P-values calculated by two-tailed unpaired Student´s t-test (b, e). ns: non-significant. Source data are provided as a Source Data file.


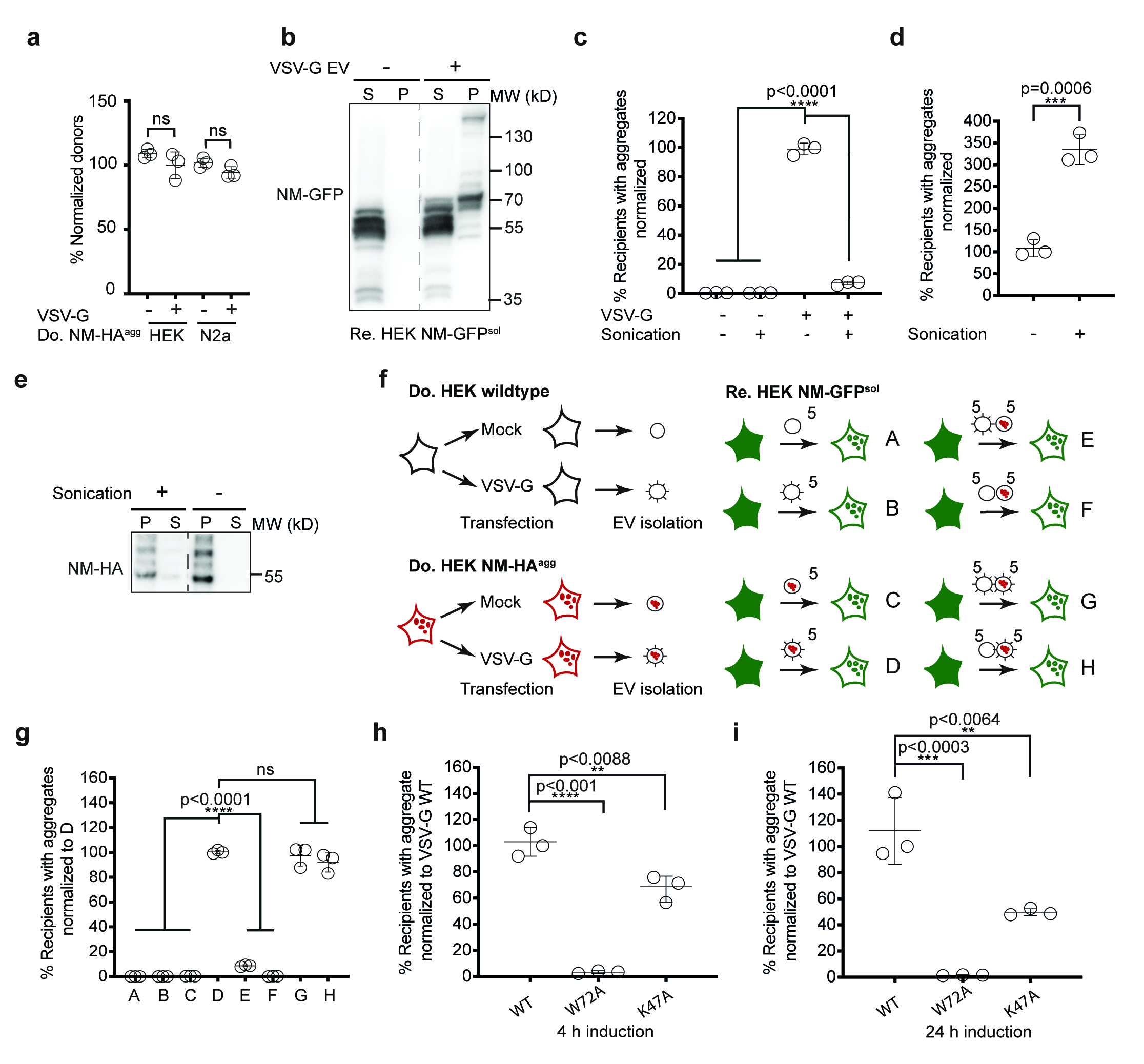


**Suppl. figure 3. Increase in NM-GFP aggregate induction is mediated by intact EV pseudotyped with fusogenic VSV-G.** a. HEK and N2a NM-HA^agg^ were transfected with Mock vector or plasmid coding for VSV-G. Total cell numbers were assessed 3 d post transfection. Mock control was used for normalization. b. Sedimentation assay of lysates from HEK NM-GFP cells exposed/ not exposed to VSV-G-pseudotyped EV from HEK NM-HA^agg^ donor cells. Additional lanes were excised for presentation purposes (dashed line). P: pellet; S: supernatant. c. Percentage of HEK NM-GFP^agg^ cells induced by VSV-G EV. Positive cells that had been exposed to VSV-G-coated, non-sonicated EV were set to 100 %. d. Percentage of HEK NM-GFP^agg^ cells induced by recombinant NM fibrils. Fibrils had been left untreated or were sonicated using the same settings. A value of 100 % represents the percentage of cells with induced aggregates exposed to non-sonicated fibrils. e. VSV-G- EV were subjected to sonication und lysates were subsequently assessed for sedimented NM-HA. Additional lanes were excised for presentation purposes (dashed line). f. Experiment to assess the effect of VSV-G in trans. Wildtype HEK cells or HEK NM-HA^agg^ donors were transfected with empty vector or vector coding for VSV-G. EV were combined or added separately to recipient HEK NM-GFP^sol^ cells in 8 combinations (A-H). Aggregate induction was determined 24 h later. g. Analysis of experiment shown in (f). Percentage of induced cells with aggregates from experiment D was set to 100 %. h., i. Effect of VSV-G mutants on EV mediated aggregate induction in HEK NM-GFP^sol^ cells. HEK NM-HA^agg^ cells were transfected with plasmids coding for wildtype VSV-G or mutants. EV were isolated 24 h later. Recipient cells were exposed to adjusted EV numbers for 4 h (h) or 24 h (i). The percentage of recipient cells with NM-GFP aggregates induced by WT VSV-G-coated EV was set to 100 %. All data are shown as the means ± SD from three (a, c, d, g-i) replicate cell cultures. Three (a, c, d, g-i) independent experiments were carried out with similar results. P-values calculated by two-tailed unpaired Student´s t-test (a, d) or one-way Anova (c, g-i). ns: non-significant. Source data are provided as a Source Data file.


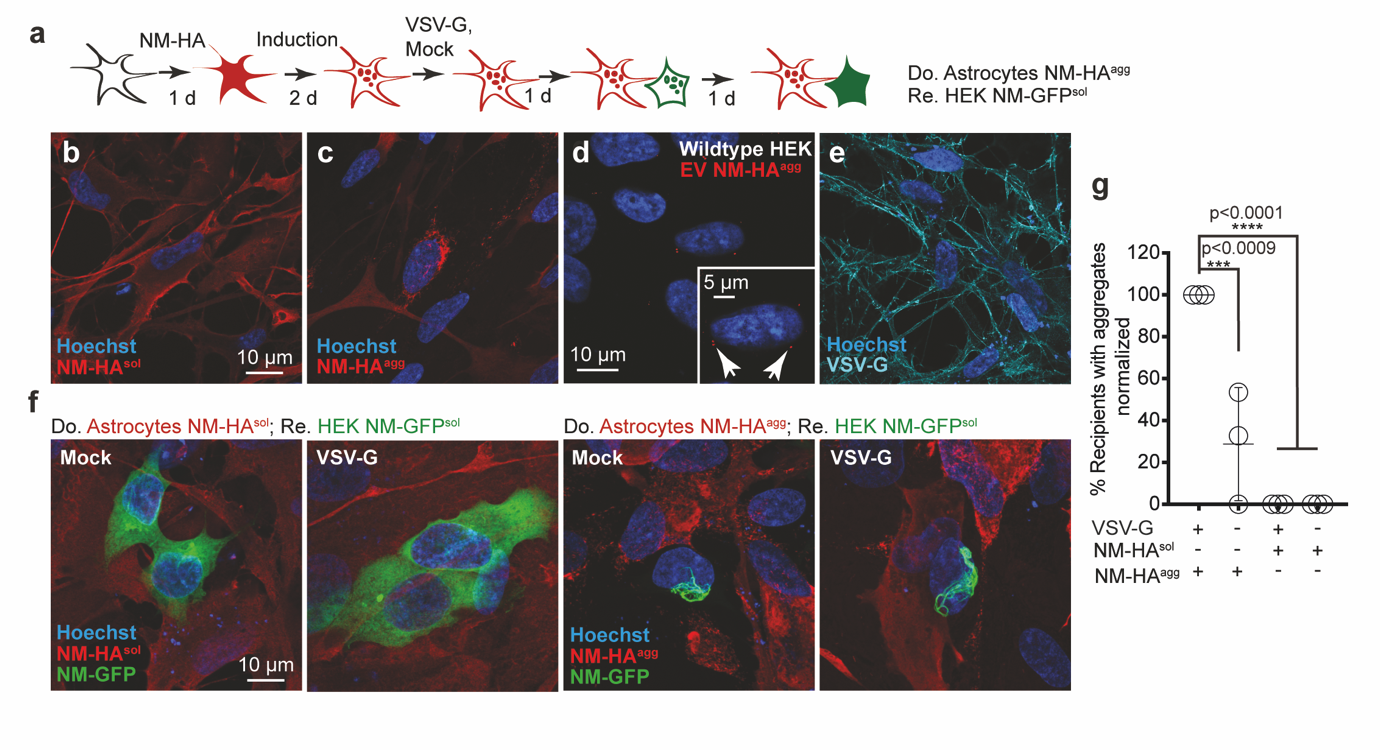


**Suppl. figure 4. Human primary astrocytes with NM-HA^agg^ induce NM-GFP aggregation in cocultured HEK NM-GFP^sol^.** a. Experimental design. b. Human primary astrocytes transduced with lentivirus coding for NM-HA. c. NM-HA aggregation upon exposure to VSV-G coated EV from HEK NM-HA^agg^ cells. EV were used to achieve necessary induction rates for cocultures. d. Control wildtype astrocytes exposed to EV from HEK NM-HA^agg^ donors with internalized NM-HA^agg^. e. Expression of VSV-G in astrocytes. f. Astrocytes with NM-HA cocultured with recipient HEK NM-GFP^sol^ cells. Astrocytes had been transduced with control or VSV-G coding virus before. NM-HA is shown in red. g. Percentage of recipient HEK cells with NM-GFP^agg^ upon coculture. The percentage of recipient HEK NM-GFP cells with induced aggregates cocultured with VSV-G expressing NM-HA^agg^ astrocytes was set to 100 %. A minimum of 360 cells per condition and experiment was analyzed. All data are shown as the means ± SD from three replicate cell cultures. Three independent experiments were carried out with similar results. P-values calculated by one-way ANOVA. Source data are provided as a Source Data file.


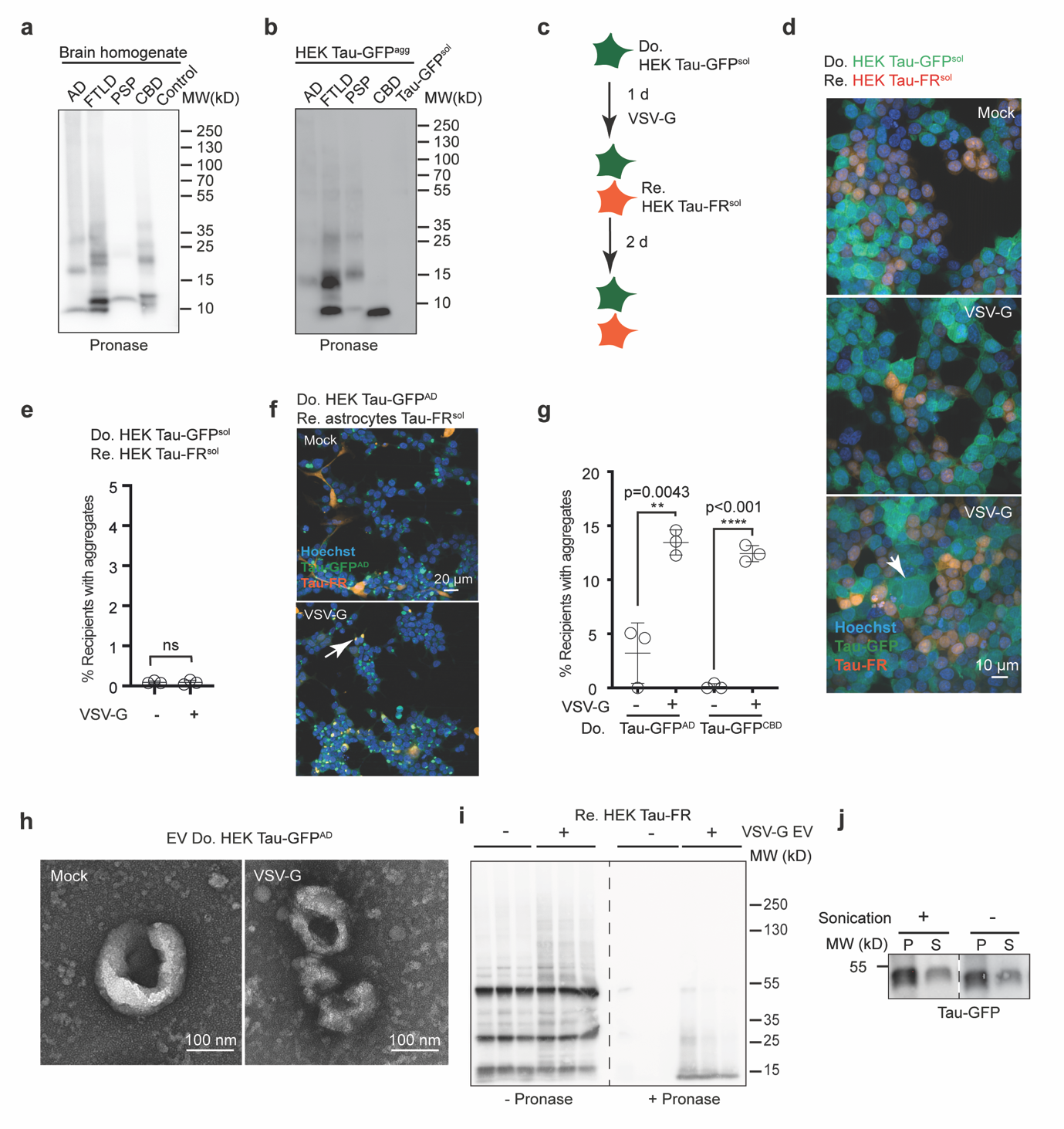


**Suppl. figure 5. Induction of Tau aggregation.** a. Different tauopathy patient brain homogenates contain Tau aggregates with distinct pronase-resistant patterns. Tau was detected using ab64193. b. Tau-GFP in HEK Tau-GFP^agg^ cells is pronase resistant. Individual HEK clones propagating Tau-GFP aggregates and control HEK Tau-GFP^sol^ cells were lysed and subjected to pronase treatment. Tau-GFP was detected using ab64193. c. Experimental design controls. Control donor HEK Tau-GFP^sol^ cells were transfected with VSV-G plasmid or Mock transfected. The following day, donor cells were cocultured with recipient HEK cells expressing Tau-FR^sol^. d. Representative images of cocultures. Arrowhead marks occasional multinucleated cells. e. Quantitative analysis of recipient cells with induced aggregates following coculture with donors expressing soluble Tau-GFP (as of (d)). f. Donor clone HEK Tau-GFP^AD^, transfected or not with VSV-G plasmid, was cocultured with human astrocytes ectopically expressing Tau-FR^sol^. g. Transfected donor clones HEK Tau-GFP^AD^ and HEK Tau-GFP^CBD^ were cocultured with human astrocytes expressing Tau-FR^sol^. Shown is the percentage of human astrocytes expressing Tau-FR^sol^ with induced Tau-FR aggregates 2 d post coculture. h. Transmission electron microscopy of EV isolated from HEK Tau-GFP^AD^ cells transfected with empty vector or VSV-G coding plasmid. i. Pronase digest of lysates from HEK Tau-FR^sol^ cells before and after cells were exposed to VSV-G coated EV. Experiments were performed in triplicates. Tau-FR was detected using anti-Tau ab64193. j. Sedimentation assay demonstrating that Tau-GFP^AD^ remains aggregated in EV fractions that were subjected to sonication. Tau-GFP was detected using ab64193. Additional lanes were excised for presentation purposes (dashed line). P: pellet; S: supernatant fraction. All data are shown as the means ± SD from three (e, g) replicate cell cultures. Three (e, g) independent experiments were carried out with similar results. P-values calculated by two-tailed unpaired Student´s t-test (e, g). ns: non-significant. Source data are provided as a Source Data file.

**References**

1. Montine TJ*, et al.* National Institute on Aging-Alzheimer's Association guidelines for the neuropathologic assessment of Alzheimer's disease: a practical approach. *Acta Neuropathol* **123**, 1-11 (2012).

2. Krammer C*, et al.* Prion protein/protein interactions: fusion with yeast Sup35p-NM modulates cytosolic PrP aggregation in mammalian cells. *FASEB J* **22**, 762-773 (2008).

3. van Driel IR, Davis CG, Goldstein JL, Brown MS. Self-association of the low density lipoprotein receptor mediated by the cytoplasmic domain. *J Biol Chem* **262**, 16127-16134 (1987).
