# Supplementary material for: Highly efficient intercellular spreading of protein misfolding mediated by viral ligand-receptor interactions
Source: Nat Commun. 2021 Oct 19;12:5739. doi: 10.1038/s41467-021-25855-2 (PMC8526834; doi:10.1038/s41467-021-25855-2)
Supplement: Supplementary file 3 — Description of Additional Supplementary Information [file 41467_2021_25855_MOESM3_ESM.pdf]

Description of Additional Supplementary Information

File Name: Supplementary Movie 1

Description: . Kinetics of EV-mediated NM-GFP aggregate induction. HEK NM-GFPsol cells were exposed to EV from HEK NM-HAagg cells transfected with VSV-G plasmid. Life cell imaging was initiated 2h after EV exposure and lasts for up to 15 h. Automated images were taken each 20 min.
