# Supplementary material for: Highly efficient intercellular spreading of protein misfolding mediated by viral ligand-receptor interactions
Source: Nat Commun. 2021 Oct 19;12:5739. doi: 10.1038/s41467-021-25855-2 (PMC8526834; doi:10.1038/s41467-021-25855-2)
Supplement: Supplementary file 5 — Reporting Summary [file 41467_2021_25855_MOESM5_ESM.pdf]

nature research

Corresponding author(s): Ina Vorberg

Last updated by author(s): Jul 20, 2021

Reporting Summary

Nature Research wishes to improve the reproducibility of the work that we publish. This form provides structure for consistency and transparency in reporting. For further information on Nature Research policies, see our Editorial Policies and the Editorial Policy Checklist.

Statistics

For all statistical analyses, confirm that the following items are present in the figure legend, table legend, main text, or Methods section.

n/a	Confirmed	
☐	☒	The exact sample size (n) for each experimental group/condition, given as a discrete number and unit of measurement
☐	☒	A statement on whether measurements were taken from distinct samples or whether the same sample was measured repeatedly
☐	☒	The statistical test(s) used AND whether they are one- or two-sided<br>*Only common tests should be described solely by name; describe more complex techniques in the Methods section.*
☒	☐	A description of all covariates tested
☒	☐	A description of any assumptions or corrections, such as tests of normality and adjustment for multiple comparisons
☐	☒	A full description of the statistical parameters including central tendency (e.g. means) or other basic estimates (e.g. regression coefficient) AND variation (e.g. standard deviation) or associated estimates of uncertainty (e.g. confidence intervals)
☐	☒	For null hypothesis testing, the test statistic (e.g. F, t, r) with confidence intervals, effect sizes, degrees of freedom and P value noted<br>*Give P values as exact values whenever suitable.*
☒	☐	For Bayesian analysis, information on the choice of priors and Markov chain Monte Carlo settings
☒	☐	For hierarchical and complex designs, identification of the appropriate level for tests and full reporting of outcomes
☒	☐	Estimates of effect sizes (e.g. Cohen's d, Pearson's r), indicating how they were calculated

Our web collection on statistics for biologists contains articles on many of the points above.

Software and code

Policy information about availability of computer code

Data collection	Automated confocal microscope Cellvoyager CV6000 (Yokogawa Inc.)<br>Confocal laser scanning microscope LSM 800 with Airyscan (Zeiss)<br>Imaging system Fusion FX (Vilber Lourmat)<br>Plate reader Fluostar Omega BMG (BMG Labtech)<br>ZetaView PMX 110-SZ-488 Nano Particle Tracking Analyzer (Particle Metrix GmbH)
Data analysis	Automated image analysis was performed with the Cellvoyager Analysis support software (Yokogawa Inc.; CV7000 Analysis Software; Version 3.5.1.18).<br>Confocal microscope images were analyzed via Zen 2010 (ZenBlue, Zeiss)<br>Plate reader data was analyzed using MARS Data Analysis Software (BMG Labtech)<br>Statistical analyses were performed using Prism 6.0 (GraphPad Software v.7.0c).

For manuscripts utilizing custom algorithms or software that are central to the research but not yet described in published literature, software must be made available to editors and reviewers. We strongly encourage code deposition in a community repository (e.g. GitHub). See the Nature Research guidelines for submitting code & software for further information.

Data

Policy information about availability of data

All manuscripts must include a data availability statement. This statement should provide the following information, where applicable:

- Accession codes, unique identifiers, or web links for publicly available datasets
- A list of figures that have associated raw data
- A description of any restrictions on data availability

All data generated or analysed during this study are included in this published article (and its data source file).

Field-specific reporting

Please select the one below that is the best fit for your research. If you are not sure, read the appropriate sections before making your selection.

[x] Life sciences [ ] Behavioural & social sciences [ ] Ecological, evolutionary & environmental sciences

For a reference copy of the document with all sections, see nature.com/documents/nr-reporting-summary-flat.pdf

Life sciences study design

All studies must disclose on these points even when the disclosure is negative.

Sample size	Sample size was determined based on empirical data from the existing publications and experience in similar studies performed previously by our laboratory and the DZNE laboratory automation facility (Duernberger et al. 2018, doi: 10.1128/MCB.00111-18).
Data exclusions	No data was excluded from analyses.
Replication	6 wells with cells were analyzed per coculture experiment (n=6). 3 wells were analyzed per EV experiment (n=3). For quantitative analysis, at least 6000 cells were analyzed. Experiments were repeated at least twice, with similar results, except for spike S pseudotyped EV (two independent experiments, with similar results) and experiments with human astrocyte EV donors (twice). Chemical inhibition experiments of EV uptake and knock-down of clathrin heavy chain: Two independent experiments, with similar results, with quantitative analysis performed after 4h and 24h.
Randomization	Randomly chosen identical cell culture populations were cocultured or were exposed to extracellular vesicles (EV) obtained from target cells or EV obtained from control cells.
Blinding	No blinding was done. Automated image acquisition and analysis was performed to minimize bias.

Reporting for specific materials, systems and methods

We require information from authors about some types of materials, experimental systems and methods used in many studies. Here, indicate whether each material, system or method listed is relevant to your study. If you are not sure if a list item applies to your research, read the appropriate section before selecting a response.

Materials & experimental systems

n/a	Involved in the study	
[ ]	[x]	Antibodies
[ ]	[x]	Eukaryotic cell lines
[x]	[ ]	Palaeontology and archaeology
[x]	[ ]	Animals and other organisms
[ ]	[x]	Human research participants
[x]	[ ]	Clinical data
[x]	[ ]	Dual use research of concern

Methods

n/a	Involved in the study	
[x]	[ ]	ChIP-seq
[x]	[ ]	Flow cytometry
[x]	[ ]	MRI-based neuroimaging

Antibodies

Antibodies used	Mouse anti-PrP antibody 4H11 (generated by Hermann Schätzl, Technical University of Munich, and Elisabeth Kremmer, Helmholtz Center Munich; 1:10)<br>rat anti-NM 4A5-1111 (Krammer et al. 2009, doi: 10.4161/pri.3.4.10013; 1:10)<br>mouse Alexa Fluor 647-conjugated anti-HA antibody (MBL, M180-A647, lot: 004; 1:500)<br>rabbit anti-Tau (Abcam, ab64193, lot: GR3244995-1; 1:1000)<br>rabbit anti-hACE2 (Abcam, ab15348, lot: GR3333640-4; 1:1000)<br>mouse anti-VSV-G (Sigma, A5977, lot: 078M4885V; 1:1000)

mouse anti-VSV-G (Kerafast, EB0010; 1:400)
mouse anti-GAPDH 6C5 (Abcam, ab8245, lot :GR3275542-4; 1:5000)
rabbit anti-Flotillin1 (Abcam, ab133497, lot: GR217473-1; 1:1000)
mouse anti-Alix (BD Bioscience, lot: 6217567; 1:1000)
mouse anti-SARS-CoV-2 spike S (GeneTex, GTX632604; 1:1000)
rat anti-HA (Roche, 3F10, lot: 42155800; 1:1000)
mouse anti-Hsc/Hsp70 (ENZO, N27F3-4, lot 09061121; 1:1000)
rabbit anti-clathrin heavy chain (Abcam, ab21679; 1:1000)
rabbit anti-GFP (Abcam, ab183734, lot: GR298298-12; 1:5000)
mouse anti-LDL receptor NBP1-78159SS (Novusbiologicals, NBP1-78159SS, lot: A-4; 1:500)
anti-mouse horseradish peroxidase (HRP)-conjugated secondary AB (Dianova, 115-035-062; 1:10.000)
anti-rat HRP-conjugated secondary AB (Dianova, 112-035-062; 1:10.000)
anti-rabbit HRP-conjugated secondary AB (Dianova, 111-035-144; 1:10.000)
Alexa Fluor 488 goat anti-rabbit IgG (Invitrogen, A11034, lot: 2018207; 1:800)
Alexa Fluor 488 goat anti-mouse IgG (Invitrogen, A11029, lot: 2066709; 1:800)

Validation

Commercially available antibodies were used according to the data sheets provided by the manufacturers for each assay.

Validation:
rabbit anti-Tau (Abcam, ab64193, lot: GR3244995-1). Suitable for WB, ICC. Reacts with mouse, zebrafish. 55 references provided by supplier (Abcam). In house validation WB of ectopically expressed Tau.
rabbit anti-hACE2 (Abcam, ab15348, lot: GR3333640-4). Suitable for WB, IHC-P. Reacts with mouse, rat, human ACE2. 35 references provided by supplier (Abcam). In house validation WB of ectopically expressed ACE2.
mouse anti-VSV-G peroxidase (Sigma, #A5977, clone P5D4, lot: 078M4885V). Suitable for WB (Sigma, 5 references provided). In-house validation by WB of ectopically expressed protein.
mouse anti-VSV-G (Kerafast, #EB0010, clone 8G5F11). Suitable for WB, flow cytometry, IF (46 references provided by Kerafast). In-house validation by IF of ectopically expressed VSV-G.
mouse anti-GAPDH (Abcam, ab8245, clone 6C5 , lot :GR3275542-4). Suitable for WB, ICC/IF (2735 references provided by Abcam). Reacts with mouse, rat, human. In-house validation by WB, detecting appropriate size protein.
rabbit anti-Flotillin1 (Abcam, ab133497, clone EPR6041, lot: GR217473-1). Suitable for WB, IHC-P, IP, ICC/IF. Knock-out validated. Reacts with mouse, rat, human. 23 references provided by Abcam). In-house validated by WB, detecting appropriate size protein.
mouse anti-Hsp70/72 (Enzo, C92F3A-5, lot 05021648). Suitable for ELISA, flow cytometry, ICC, IF, IHC, IP, WB, electron microscopy. Reacts with human, rat, mouse and others. In-house validation by WB, detects appropriate size protein.
mouse anti-Alix (BD Bioscience, lot: 6217567)
mouse anti-SARS-CoV-2 spike S (GeneTex, #GTX632604, clone 1A9). Suitable for WB, ICC/IF, IHC-P, FACS, IP, ELISA, EM, Sandwich ELISA, IHC-P. GeneTex provides 84 references. Reacts with ARS Coronavirus-2. In-house validation by WB of ectopically expressed spike S.
rat anti-HA (Roche, 3F10, lot: 42155800). In-house validation by WB of ectopically expressed HA-tagged proteins.
mouse anti-Hsc/Hsp70 (ENZO, N27F3-4, lot 09061121). Reacts with human, mouse, rat and others. Suitable for Flow Cytometry, IHC (PS), IP, WB. In-house validation by WB detecting correct size protein.
rabbit anti-clathrin heavy chain (Abcam, ab21679). Suitable for WB, ICC. Reacts with mouse, rat, human. Abcam provides 94 references. In-house validation by knock-down and WB.
rabbit anti-GFP (Abcam, ab183734, clone, EPR14104lot: GR298298-12). Reacts with Aequorea victoria GFP. Suitable for Flow Cytometry (Intra), WB, IHC-P, ICC/IF. Abcam provides 39 references. In-house validation by IF of ectopically expressed GFP-fusion proteins.
mouse anti-LDL receptor NBP1-78159SS (Novusbiologicals, clone C7, NBP1-78159SS, lot: A-4). Suitable for WB, ELISA, EM, Flow, ICC/IF, IHC, IHC-Fr, IHC-P, In vitro, IP, PLA, RIA. Provider lists 20 references. In-house validation by detection of appropriate protein bands by WB.

Anti-PrP antibody 4H11 was generated and validated by Ertmer et al., Institute of Virology, Prion Research Group, Technical University of Munich. The details can be found in the following article: A.Ertmer, S.Gilch et al. The Tyrosine Kinase Inhibitor STI571 Induces Cellular Clearance of PrPSc in Prion-infected Cells. The Journal of Biological Chemistry. DOI 10.1074/jbc.M405652200. We confirmed that the antibody does not detect PrP in PrP knock-out cells (Maas et al., The Jounral of Biological Chemistry. DOI: 10.1074/jbc.M701309200).

Antibody anti-NM was generated and validated by us (Krammer et al., PNAS 2009). The antibody was raised against peptide IKEQEEEVDDEV comprising amino acid residues 239-250 in the M domain of Saccharomyces cerevisiae Sup35 (DOI: 10.1073/pnas.0811571106). The antibody was in-house validated by IF and WB upon ectopic expression of Sup35 NM.

Uncropped gel images are provided with the manuscript source data.

Eukaryotic cell lines

Policy information about cell lines

Cell line source(s)

HEK 293T, Neuro-2a, and L-929 cells were purchased from ATCC.
Vero cells were purchased from CLS (Cell lines service).
CAD5 cells were kindly provided by Corinne Lasmezas, Scripps Research Institute, 10550 North Torrey Pines Road, TPC9, La Jolla, California 92037, USA. CAD is a central nervous system catecholaminergic cell line originally established by Qi et al. (1997): https://doi.org/10.1523/JNEUROSCI.17-04-01217.1997. The scrapie-susceptible subclone CAD5 was established by

	Mahal et al. (2007): https://doi.org/10.1073/pnas.0710054104.<br>Human astrocytes (Cat No.: 1800; Lot No.:25536) were purchased from ScienCell Research Laboratories.
Authentication	All cell lines from ATCC and CLS were authenticated via Short tandem repeat (STR) profiling.<br>CAD5 cells were authenticated by their susceptibility to prion infection, as shown by S. Mahal et al. (2007). Cells were cloned by the Charles Weissmann lab for high susceptibility to prions.
Mycoplasma contamination	All cell lines were regularly tested for mycoplasma using PCR Mycoplasma Test Kit II A8994 (PanReac AppliChem), lot 11151118. Cell lines were free of mycoplasma contamination.
Commonly misidentified lines<br>(See ICLAC register)	None.

Human research participants

Policy information about studies involving human research participants

Population characteristics	Frozen postmortem brain tissue was obtained from Brain Bank cases with a neuropathological diagnosis of CBD, PSP, FTLD-tau, Alzheimer's disease, as well as controls with absence of neurodegenerative disease. Neuropathological diagnoses was performed by a trained neuropathologist according to current consensus criteria and guidelines including scoring of Alzheimer disease neuropathological changes (ABC score) according to the National Institute of Aging-Alzheimer's association guidelines. Postmortem brain samples were obtained from 5 cases (3 male, 2 female). Details (NP diagnosis, sex, age at death, ABC score, brain region) were as follows:<br>Alzheimer's disease (AD), male, 65 y, ABC score A3, B3, C3, frontal cortex.<br>Corticobasal degeneration (CBD), female, 63 y, ABC score A2, Bx, C1, frontal cortex.<br>Progressive supranuclear palsy (PSP), male, 77 y, ABC score A1, B1, C1, pons.<br>Frontotemporal lobar degeneration-tau (FTLD-tau, MAPT IVS10+3 G>A)), male, 56 y, ABC score A0, Bx, C0, frontal cortex.<br>Control, female, 79 y, ABC score A1, B1, C0, frontal cortex.
Recruitment	Cases and regions were selected based on the presence/abundance of characteristic tau pathology (for tauopathies) or absence of tau pathology (control).
Ethics oversight	Research has been performed in accordance with the Declaration of Helsinki. Frozen postmortem brain tissue samples from neuropathologically confirmed cases of AD, FTLD-tau, CBD, PSP and control were provided by the Brain Bank associated with the University Hospital and DZNE Tübingen. In this Brain Bank, material and data is sampled and collected from donors upon written informed consent for brain autopsy and the use of the material and clinical information for research purposes obtained by the probands or their legal representative according to approval of the responsible ethic committee ("Ethik-Kommission, Medizinischen Fakultät der Eberhard-Karls-Universität und am Universitätsklinikum Tuebingen" IEC project no: 252/2013B01 and 386/2017BO1). Ethical approval for use of human samples for the current study was obtained from "Medizinische Fakultät Ethik-Kommission, Rheinische Friedrich-Wilhelms-Universität, project no. 236/18 (2018)".

Note that full information on the approval of the study protocol must also be provided in the manuscript.
